# Using Complementary Ensemble Empirical Mode Decomposition and Gated Recurrent Unit to Predict Landslide Displacements in Dam Reservoir

**DOI:** 10.3390/s22041320

**Published:** 2022-02-09

**Authors:** Beibei Yang, Ting Xiao, Luqi Wang, Wei Huang

**Affiliations:** 1 School of Engineering, Yantai University, Yantai 264005, China; yangbeibei@ytu.edu.cn; 2 School of Geosciences and Info-Physics, Central South University, Changsha 410083, China; 3 School of Civil Engineering, Chongqing University, Chongqing 400044, China; wlq93@cqu.edu.cn; 4 The Seventh Geological Brigade of Hubei Geological Bureau, Yichang 443000, China; huangwei@cug.edu.cn

**Keywords:** reservoir landslide, displacement prediction, time series analysis, complementary ensemble empirical mode decomposition, gated recurrent unit

## Abstract

It is crucial to predict landslide displacement accurately for establishing a reliable early warning system. Such a requirement is more urgent for landslides in the reservoir area. The main reason is that an inaccurate prediction can lead to riverine disasters and secondary surge disasters. Machine learning (ML) methods have been developed and commonly applied in landslide displacement prediction because of their powerful nonlinear processing ability. Recently, deep ML methods have become popular, as they can deal with more complicated problems than conventional ML methods. However, it is usually not easy to obtain a well-trained deep ML model, as many hyperparameters need to be trained. In this paper, a deep ML method—the gated recurrent unit (GRU)—with the advantages of a powerful prediction ability and fewer hyperparameters, was applied to forecast landslide displacement in the dam reservoir. The accumulated displacement was firstly decomposed into a trend term, a periodic term, and a stochastic term by complementary ensemble empirical mode decomposition (CEEMD). A univariate GRU model and a multivariable GRU model were employed to forecast trend and stochastic displacements, respectively. A multivariable GRU model was applied to predict periodic displacement, and another two popular ML methods—long short-term memory neural networks (LSTM) and random forest (RF)—were used for comparison. Precipitation, reservoir level, and previous displacement were considered to be candidate-triggering factors for inputs of the models. The Baijiabao landslide, located in the Three Gorges Reservoir Area (TGRA), was taken as a case study to test the prediction ability of the model. The results demonstrated that the GRU algorithm provided the most encouraging results. Such a satisfactory prediction accuracy of the GRU algorithm depends on its ability to fully use the historical information while having fewer hyperparameters to train. It is concluded that the proposed model can be a valuable tool for predicting the displacements of landslides in the TGRA and other dam reservoirs.

## 1. Introduction

Landslides are one of the most catastrophic disasters and are widely distributed in numerous parts of the world [1,2,3,4]. In China, annual reports from China Institute of Geo-Environment Monitoring (IGEM) show that landslides account for more than 50% of all geological hazards in recent years [5]. In 2020, for instance, 7840 geology-related hazards occurred in China, resulting in 139 deaths or people missing, 58 people injured, and a direct economic loss of CNY 5.02 billion. Among these geological disasters, 4810 were landslides, accounting for 61.3% of the total. Other types of hazards in 2020 included 1797 avalanches, 899 debris flows, 183 ground collapses, 143 ground fissures, and 8 cases of ground subsidence.

**Figure 1:**
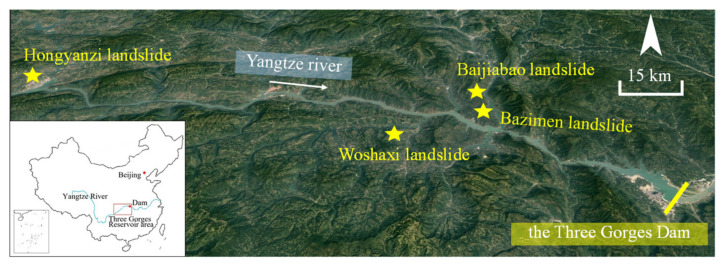
Location map of landslides in TGRA mentioned in the paper.

As one of the most landslide-prone areas in China, the Three Gorges Reservoir Area (TGRA) has been given much attention concerning severe landslides [6]. One main reason is that the construction of the Three Gorges Dam (TGD) has significantly changed the regional hydrogeological conditions [7,8]. Some landslides in the TGRA (e.g., Bazimen landslide) have deformed continuously for several decades, whereas some landslides (e.g., Woshaxi landslide) have achieved a displacement of 28,065.9 mm, and the deformation is still increasing [9,10]. Once landslides in dam reservoirs occur, they can cause severe damage along both sides of the reservoir area. In addition, these reservoir landslides can induce secondary surge disasters, endangering the shipping and bridges along the river and its tributaries [11]. The Honyanzi landslide, which occurred on 24 June 2015, was such an example, initiating a reservoir tsunami that resulted in two deaths and severe damage to shipping facilities (Figure 1) [12]. These risks can be mitigated if one can establish reliable early warning systems. As landslide displacement can represent its evolution intuitively, accurate landslide displacement prediction is an effective means of establishing such reliable early warning systems [10,13,14].

In situ displacement monitoring techniques have been available since the 1940s, especially the global positioning system (GPS) technique [15,16,17]. These techniques make it possible to acquire real-time monitoring information. These monitoring data have been applied extensively in landslide displacement prediction (LDP). The research of LDP dates back to the 1960s with the presentation of the Saito model. Subsequently, numerous LDP theories and models have been successively proposed [18]. The development of LDP research can be summarized into three stages [14,19,20]. The first stage (from the 1960s to 1970s) is the phenomenological and empirical prediction, mainly based on the macroscopic deformation phenomenon before landslide failure. The prediction accuracy is usually unsatisfied because of a high dependence on the gained experience. The second stage (during the 1980s) is the displacement-time statistical analysis prediction, leading qualitative prediction to quantitative prediction. Benefiting from the development of mathematical sciences, various statistical mathematical models have been proposed and applied to the LDP (e.g., grey system theory) [21]. Without considering influencing factors, these models are built from statistics and mathematics. Hence, these approaches are primarily valid for landslides with similar deformation characteristics [22]. The third stage (from the 1990s to the present) is the nonlinear prediction and intelligent integrated prediction. Numerous nonlinear and intelligent LDP models have been proposed and applied in cases. These models can build relationships between landslide displacement and multiple triggering factors. Their prediction performance has shown encouraging improvement.

As intelligent algorithms, machine learning (ML) models have been extensively utilized to predict landslide displacements because of their nonlinear processing ability. These models, such as the back-propagation (BP) neural network [23,24], extreme learning machine (ELM) [25,26,27,28,29], random forest (RF) [30,31], and support vector machine (SVM) [32,33,34], have become popular and have been adopted in some landslide cases in the TGRA. Influencing factors and displacement are set as the input and output of the models, respectively. The trained models have achieved encouraging performances. Zhou et al. [27] selected an artificial bees colony (ABC) to optimize the parameters of a kernel-based extreme learning machine (KELM) for LDP. Li et al. [28] proposed an ensemble-based ELM and copula model to predict the displacement of the Baishuihe landslide in the TGRA. Hu et al. [30] developed an integrated LDP model by combining the Verhulst inverse function (VIF) and RF algorithm, which provided a practical approach for predicting the long-term deformation of landslides. Bui et al. [34] adopted ABC optimization to model the least squares support vector regression (LSSVR). These forecasting models belong to static models, whereas the evolution of landslides is a complex nonlinear dynamic process [35]. The deformation conditions of landslides at one time can be affected by that of the former time [36]. A dynamic model—long short-term memory (LSTM) neural networks—was applied to LDP [9]. Jiang et al. [37] combined the support vector regression (SVR) algorithm and LSTM model to forecast the displacement of the Shengjibao landslide in the TGRA. As a deep ML method, LSTM can deal with more complicated time series predictions. With the increment of the number of available monitoring data and the improvements in computer hardware and software, the LSTM model has become a priority choice to deal with more complicated time series prediction [38,39]. One drawback of LSTM is that it has more parameters to be trained than classical ML methods, which makes it challenging to obtain the optimum of all parameters simultaneously [10]. An improved version of the LSTM—the gated recurrent unit (GRU)—is proposed and adopted in LDP. GRU replaced the three gates (input gate, forget gate, and output gate) of LSTM with two new gates (reset gate and update gate). This structure of GRU makes it possible to reduce the number of hyperparameters required for training. Thus, it can be easier for GRU to obtain a well-trained model than the LSTM [31].

In general, the LDP in the dam reservoir involves decomposing the total displacement into several components (trend term, periodic term, and stochastic term) according to time series analysis and then through predicting each component by different methods. Each displacement component has clear mathematical and physical significance. This treatment of LDP has been proven to be effective in previous studies [10,23,31,33,36,40,41,42]. Several decomposition methods have been adopted, such as the average moving method [10,33], double exponential smoothing [10], variational mode decomposition (VMD) [40], empirical mode decomposition (EMD) [37], ensemble empirical mode decomposition (EEMD) [40], and wavelet transform (WT) [41]. It is critical to forecast periodic displacement accurately to ensure the good prediction performance of accumulated displacement for landslides [23]. The prediction of periodic displacement is a heated topic, and the predictive models are summarized as mentioned above. The trend displacement is usually modeled and predicted by fitting the curve of displacement–time with polynomial functions [23,31,33]. A piecewise curve may need several polynomial functions [10]. Another displacement component—the stochastic term—is usually ignored [10,32,37,43]. The main reason is that stochastic displacement is influenced by varied, ever-present, and unquantifiable stochastic factors.

This paper decomposed accumulated displacement into a trend term, periodic term, and stochastic term by CEEMD. A univariate and a multivariable GRU model were used to predict the trend and stochastic displacements, respectively. A multivariable GRU model was adopted to predict periodic term displacement, and another two popular ML methods—LSTM and RF—were used for comparison. The proposed model was applied in the displacement prediction of the Baijiabao landslide in the TGRA. The deep dynamic model has the advantages of a powerful prediction ability with a simpler structure and fewer trained hyperparameters. In addition, the stochastic displacement, neglected in most exiting prediction models, was considered in the proposed model.

## 2. Approach to Model Displacements in Three Gorges Dam Reservoir

### 2.1. Time Series Decomposition

The change in landslide accumulated displacement is determined by geological conditions, triggering factors, and stochastic factors [10,33]. Geological conditions involve internal factors, such as the geological structure, topography, lithology, etc. Triggering factors for landslides in the TGRA are mainly the seasonal rainfall and reservoir level fluctuation. Stochastic factors appear with uncertainties, including earthquakes, traffic load, wind load, etc. The displacement components induced by the above three factors can be represented as trend displacement, periodic displacement, and stochastic displacement, respectively. Consequently, the accumulated displacement can be expressed as Equation (1):

A = T + P + S(1)

where A is accumulated displacement, T is trend displacement, P is periodic displacement, and S is stochastic displacement.

### 2.2. Complementary Ensemble Empirical Mode Decomposition

Empirical mode decomposition (EMD) was firstly proposed by Huang et al. [44]. They implemented EMD by converting a nonlinear sequence into a set of stationary sequences that consisted of several intrinsic mode functions (IMFs) and a residual. EMD, however, has the disadvantage of mode mixing, and thus ensemble empirical mode decomposition (EEMD) was presented by Wu et al. [45]. In EEMD, uncorrelated finite white noise is added into the original signal, and the final IMF is obtained by averaging all the IMFs. Due to the dependence of the added noise in EEMD, Yeh et al. [46] presented a modified algorithm of EEMD named complete ensemble empirical mode decomposition (CEEMD) to decompose the signal into different scale IMFs. By adding opposite random white noise into the decomposition results of EEMD, CEEMD realized the advantages of an improved decomposition, better denoising, and higher computational efficiency. The following steps settle the process of CEEMD decomposing the original time series.

The first step is to add positive and negative white noise pairs to the original time series.

(2) $$\left[\begin{array}{c} B_{i}\left(t\right)\\ C_{i}\left(t\right) \end{array}\right]=\left[\begin{array}{cc} 1 & 1\\ 1 & -1 \end{array}\right]\left[\begin{array}{c} S_{i}\left(t\right)\\ a_{i}\left(t\right) \end{array}\right]$$

where $B_{i}\left(t\right)$ and $C_{i}\left(t\right)$ are the time series after adding positive and negative white noise, respectively, $S_{i}\left(t\right)$ is the original time series, and $a_{i}\left(t\right)$ is the added white noise.

Subsequently, the EMD algorithm is used to decompose $B_{i}\left(t\right)$ and $C_{i}\left(t\right)$.

(3) $$\left\{\begin{array}{c} B_{i}\left(t\right)=\overset{J}{\underset{j}{\sum }}IMF_{ij}^{+}\\ C_{i}\left(t\right)=\overset{J}{\underset{j}{\sum }}IMF_{ij}^{-} \end{array}\right.$$

where *J* is the number of *IMF* after decomposing, and $IMF_{ij}^{+}$ and $IMF_{ij}^{-}$ are the *j*th components of IMF after adding positive and negative white noise, respectively.

*N* sets of *IMFs* can be obtained after repeating the above two steps.

(4) $$\left\{\begin{array}{c} \left\{\left\{IMF_{1j}^{+},IMF_{2j}^{+},\cdots ,IMF_{Nj}^{+}\right\}\right\}\\ \left\{\left\{IMF_{1j}^{-},IMF_{2j}^{-},\cdots ,IMF_{Nj}^{-}\right\}\right\} \end{array}\right\}$$

We can obtain the final *j*th *IMF* by averaging its positive and negative components.

(5) $$IMF_{j}=\frac{1}{2N}\overset{N}{\underset{i=1}{\sum }}\left(IMF_{ij}^{+}+IMF_{ij}^{-}\right)$$

Finally, the time series $S_{i}\left(t\right)$ is decomposed as Equation (6):

(6) $$S\left(t\right)=\overset{N}{\underset{j=1}{\sum }}IMF_{j}$$

### 2.3. Machine Learning Methods

#### 2.3.1. Long Short-Term Memory Neural Network

**Figure 2:**
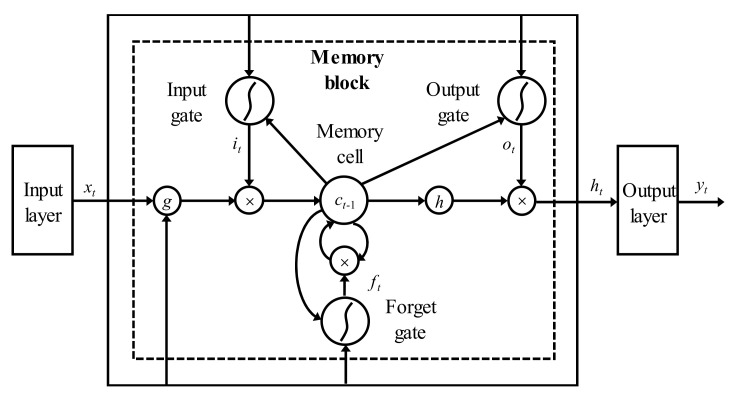
Architecture of LSTM neural network.

Long short-term memory (LSTM) neural networks are in the category of dynamic recurrent neural networks (RNN). Due to the issues of gradient vanishing and gradient exploding in conventional RNN, they cannot handle the dependency of a long time series. To avoid such disadvantages of conventional RNN, Hochreite and Schmidhuber [47] proposed LSTM in 1997. In LSTM, a memory block is used as the basic unit of its hidden layer, consisting of a memory cell and three gates, named the input gate, forget gate, and output gate (Figure 2) [48].

The input gate controls the flow of input activations into the memory cell. The information from the hidden state at step t − 1 (*h_t-1_*) and the current input value (*x_t_*) is firstly passed along to the sigmoid function (σ). Then, the information of input data from the current step and previous data from the last step is used to update and generate a new vector. The forget gate is responsible for filtering information by means of passing along useful information to the next step and abandoning useless information. The output gate controls the transfer of useful information into other memory blocks.

We recorded the input sequence as *x* = (*x*_1_, *x*_2_,…, *x_T_*), and can obtain the output sequence *y* = (*y*_1_, *y*_2_,…, *y_T_*) by treating Equation (7) to Equation (12).

(7) $$i_{t}=\sigma \left(W_{xi}x_{t}+W_{hi}h_{t-1}+W_{ci}c_{t-1}+b_{i}\right)$$

(8) $$f_{t}=\sigma \left(W_{xf}x_{t}+W_{hf}h_{t-1}+W_{cf}c_{t-1}+b_{f}\right)$$

(9) $$c_{t}=f_{t}c_{t-1}+i_{t}\tan h\left(W_{xc}x_{t}+W_{hc}h_{t-1}+b_{c}\right)$$

(10) $$O_{t}=\sigma \left(W_{xo}x_{t}+W_{ho}h_{t-1}+W_{co}c_{t-1}+b_{o}\right)$$

(11) $$h_{t}=o_{t}\tan h\left(c_{t}\right)$$

(12) $$y_{t}=W_{hy}h_{t}+b_{y}$$

where $i_{t}$, $f_{t}$, $o_{t}$, and $c_{t}$ are the values of the input gate, forget gate, output gate, and a memory cell at time t; $b_{i}$, $b_{f}$, $b_{o}$, and $b_{c}$ are their corresponding bias values; $W_{x}$ are the weights between input nodes and hidden nodes; $W_{h}$ are the weights between hidden nodes and cell memory; $W_{c}$ are the weights connecting the memory cell to output nodes; σ is the sigmoid activation function; tanh is the hyperbolic tangent function mapping data to [−1, 1]; and $h_{t}$ is the hidden state, containing information about the history of earlier elements in the series.

#### 2.3.2. Gated Recurrent Unit

**Figure 3:**
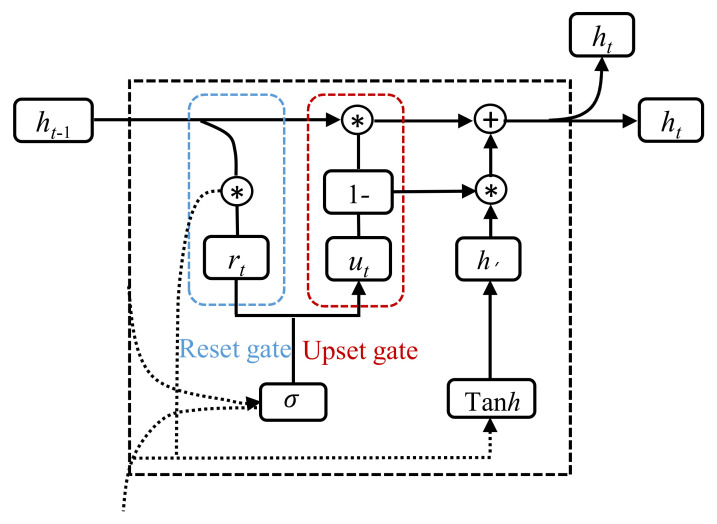
Structure chart of GRU.

The gated recurrent unit (GRU) is an improved version of LSTM. Compared with LTSM, GRU has the advantages of fewer hyperparameters and faster training by using two new gates (update gate and reset gate) (Figure 3). These two gates are utilized to store as much information as possible for a long time series [49,50]. The reset gate is responsible for determining how much information at the previous moment is passed along, and resets the information at the current moment. The update gate controls the extent of information from both the previous time step and the current time step that will be passed along to the memory cell. The equations in GRU are given as follows:

(13) $$u_{t}=\sigma \left(W_{xu}x_{t}+W_{hu}h_{t-1}+b_{u}\right)$$

(14) $$r_{t}=\sigma \left(W_{xr}x_{t}+W_{hr}h_{t-1}+b_{r}\right)$$

(15) $$h^{\prime }=\tan h\left(W_{xh}x_{t}+(r_{t}⊙h_{t-1}\right)W_{hh}+b_{h})$$

(16) $$h_{t}=\left(1-u_{t}\right)⊙h^{\prime }+u_{t}⊙h_{t-1}$$

where *u*_t_ and *r*_t_ are the values of the upset gate and reset gate, respectively; $h^{\prime }$ is the value after resetting; *W* and *b* are the weights and deviations, respectively; ⊙ represents pointwise multiplication between tensors. Other parameters indicate the same meaning as those in LSTM.

#### 2.3.3. Random Forest

Random Forest (RF) is an ensemble ML method that has been well-developed for classification, regression, and other tasks [51]. This method has some advantages, including great robustness, data adaptability, and low overfitting [52]. The RF algorithm is realized based on multiple decision trees by sampling from the original dataset (both samples and their features) [53].

To build a decision tree, we divide the predictor space into the number of *J* regions that are distinct and non-overlapping and represented as *R*_1_, …, *R_j_*. The division is implemented by minimizing the root of the sum of squares.

(17) $$\overset{J}{\underset{j=1}{\sum }}\underset{i\in R_{j}}{\sum }{\left(y_{i}-{\hat{y}}_{R_{j}}\right)}^{2}$$

where $y_{i}$ is the observation belonging to *R_j_*, and ${\hat{y}}_{R_{j}}$ is the mean response for the training observations within the *j*th region.

Bagging is used to select training sets from the original dataset, and each training set is utilized for building a decision tree. The final prediction result ${\hat{y}}_{bag}$ can be achieved by averaging the results of all decision trees (Equation (18)), which can improve the prediction accuracy by doing so.

(18) $${\hat{y}}_{bag}=\frac{1}{M}\overset{M}{\underset{i=1}{\sum }}{\hat{y}}_{i}$$

where ${\hat{y}}_{i}$ is the prediction result of the *i*th decision tree and *M* is the number of decision trees.

### 2.4. Prediction Process with the Proposed Model

**Figure 4:**
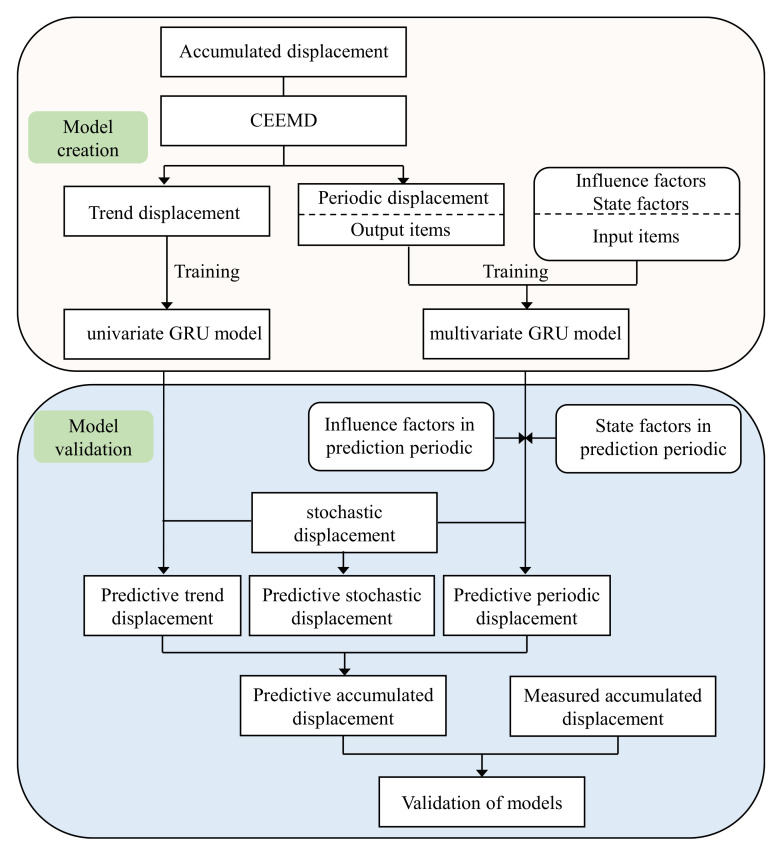
Flowchart of the proposed predictive model.

In the establishment of the proposed model (Figure 4), we adopted CEEMD to decompose the monitored accumulated displacement into a trend component and a periodic component. Subsequently, we used a univariate GRU model and a multivariate GRU model to predict the trend term and periodic term, respectively. The univariate GRU model described the trend displacement versus time, whereas the multivariate GRU model described the relationships between periodic displacement and influencing factors. A multivariate LSTM model and a multivariate RF model were also utilized for forecasting periodic displacement to verify the prediction performance of the GRU model. We adopted a multivariate GRU model to predict stochastic displacement.

The error analysis introduces the root mean square error (*RMSE*), mean absolute percentage error (*MAPE*), and the goodness of fit (*R*^2^) for validations. Smaller values of *RMSE* and *MAPE* and a larger value of *R*^2^ reflect a better prediction performance.

(19) $$RMSE=\sqrt{\frac{1}{N}\overset{N}{\underset{i=1}{\sum }}{\left(x_{i}-{\hat{x}}_{i}\right)}^{2}}$$

(20) $$MAPE=100\%\times \frac{1}{N}\overset{N}{\underset{i=1}{\sum }}\left|\frac{x_{i}-{\hat{x}}_{i}}{x_{i}}\right|$$

(21) $$R^{2}=1-\frac{N\sum {\left(x_{i}-{\hat{x}}_{i}\right)}^{2}}{N\sum x_{i}^{2}-\sum {\hat{x}}_{i}{}^{2}}$$

where $x_{i}$ and ${\hat{x}}_{i}$ represent the *i*th observed displacement and predicted displacement, respectively, and *N* is the record number of displacement.

## 3. Baijiabao Landslide Case Study

### 3.1. Overview of the Baijiabao Landslide

#### 3.1.1. Geological Conditions

**Figure 5:**
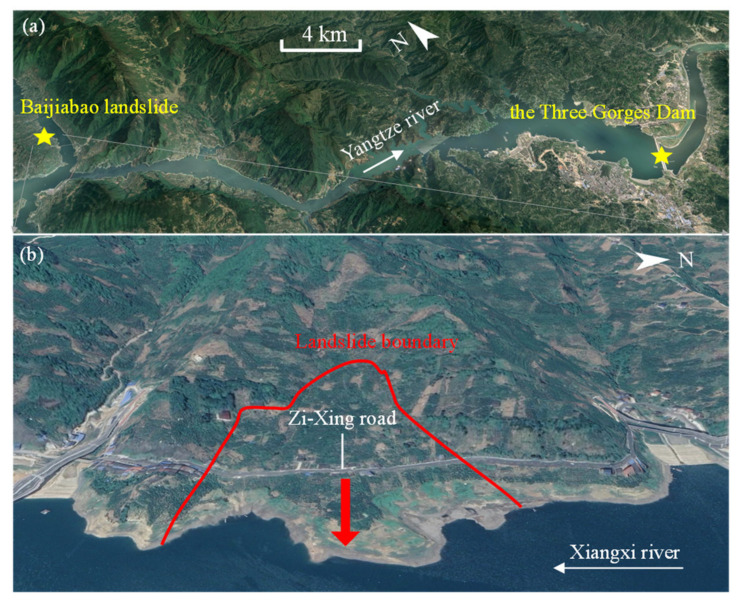
(**a**) Location of the Baijiabao landslide; (**b**) overall view of the Baijiabao landslide.

**Figure 6:**
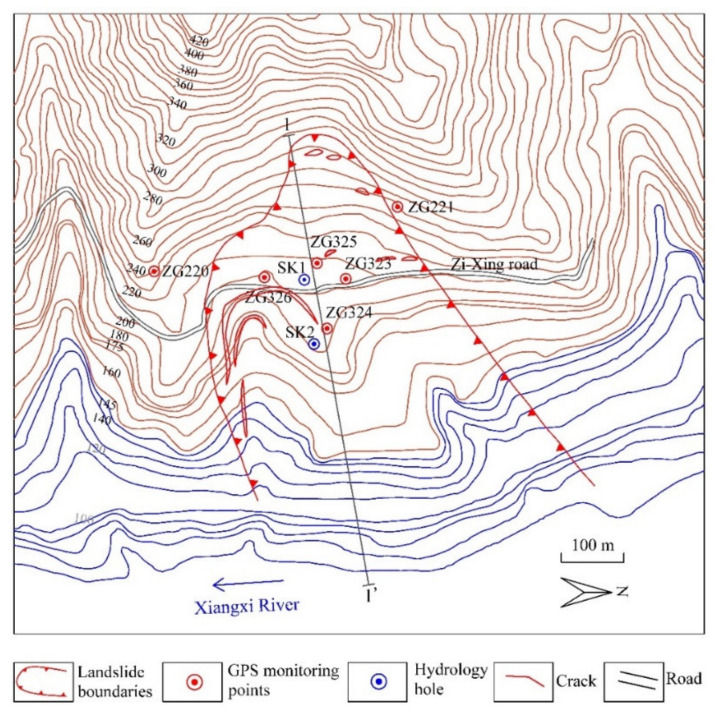
Monitoring arrangement in the Baijiabao landslide.

The Baijiabao landslide is located on the west bank of the Xiangxi River and belongs to Zigui County, Hubei Province, China (Figure 5). The Xiangxi River is a major tributary of the Yangtze River, approximately 2.5 km upstream from the estuary. The main sliding direction of the landslide is perpendicular to the Xiangxi River and orientated at N 82° E. The front part of the landslide is submerged in the Xiangxi River, whereas the interface between bedrock and soil bounds the upper edge. The left and right boundaries are defined by seasonal homologous gullies (Figure 6). The landslide has a leading-edge elevation of 160–175 m, a trailing-edge elevation of 265 m, a width of approximately 550 m, a length of approximately 400 m, an average thickness of 45 m, and an estimated volume of 9.9 × 10^6^ m^3^ [25].

**Figure 7:**
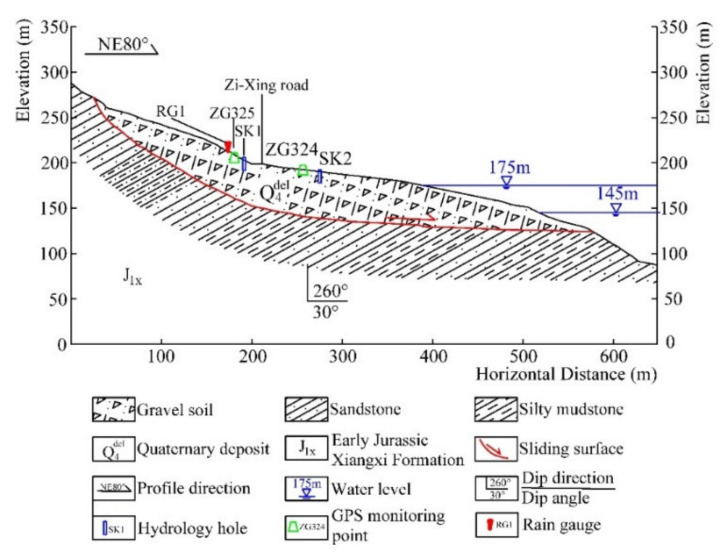
Schematic geological cross-section A–1′ of the Baijiabao landslide.

The sliding mass is mainly composed of silty clay and fragmented rubble. These sliding materials form a loose and disordered structure of the slope. The slip bed is silty mudstones and muddy siltstones of the Jurassic Xiangxi group, which dig into the hill by a direction of 260° with an angle of 30° [9]. The sliding surface is defined by the interface between colluvial materials and subjacent bedrock. The sliding zone is mainly composed of silty clay (Figure 7).

The Baijiabao landslide experienced large deformations since the impoundment of the Three Gorges Dam (TGD) in 2003 and kept deforming in the following years. In June 2007, tensile cracks with a length of 160 m and depth of 10 cm occurred at both side boundaries of the landslide close to the trailing edge. In May 2009, tensile cracks were observed on the road in the front and right parts of the landslide. A similar road deformation appeared in the middle of the landslide. In June 2012, cracks of the trailing edge showed a connecting tendency. Besides, cracks of the boundaries extended to the front part of the landslide. In June 2015, several tensile cracks, both on the right boundary and Zi-Xing road, became larger. Before the impoundment of the TGD, 165 residents used to live in the landslide area, whereas now, only 20 residents live there.

#### 3.1.2. Monitoring Data and Deformation Characteristics of the Landslide

**Figure 8:**
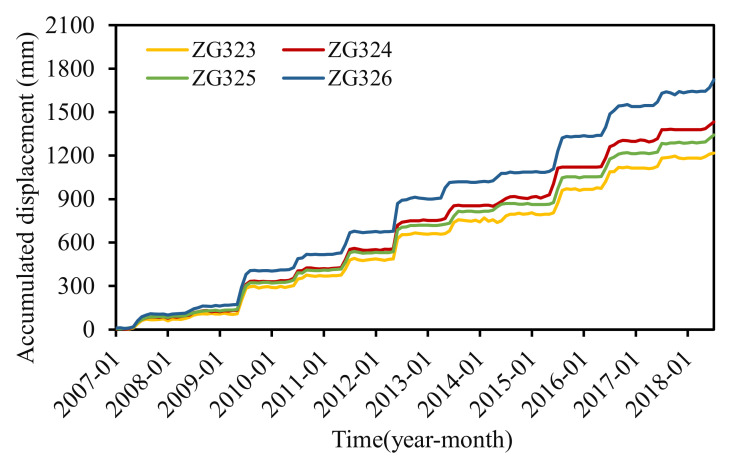
Accumulated displacement in the Baijiabao landslide.

Four GPS stations numbered ZG323, ZG324, ZG325, and ZG326 were installed in the landslide area to monitor the surface displacements at one time per month since late 2006. Another two stations numbered ZG320 and ZG321 were established as the datum stations. Monitoring data from January 2007 to July 2018 were acquired (Figure 8). The displacements of the four monitoring stations showed a similar trend of step-wise, which meant that the landslide deformed distinctly in steps during April and September (especially from May to July) and became unremarkable in other times of the year.

**Figure 9:**
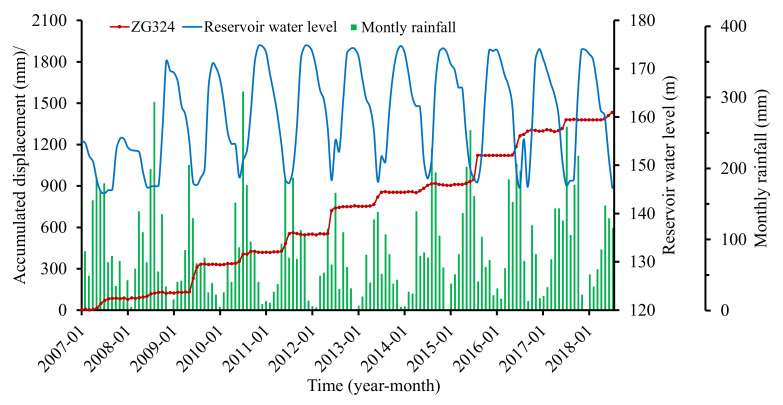
Rainfall, reservoir water level, and accumulated displacement at ZG324, Baijiabao landslide.

**Figure 10:**
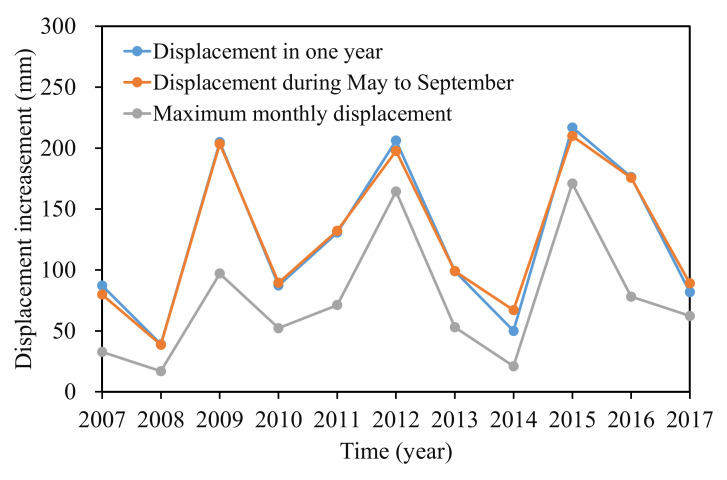
Annual displacement increment, displacement during step-wise deformation period, and the maximum monthly displacement at ZG324, Baijiabao landslide.

Cao et al. [25] analyzed the deformation characteristics and evolution of the Baijiabao landslide. The analysis showed that the Baijiabao landslide deformed as an entity. Station ZG324, located in the central position of the landslide, was chosen as a representative for establishing the displacement forecasting model. Figure 9 displayed the accumulated displacements at station ZG324, monthly rainfall, and reservoir water level, and all the data were obtained by measurement. The annual displacement, displacement during step-wise deformation period (from May to September), and the maximum monthly displacement were summarized in Figure 10.

It can be seen that a sharp displacement increment occurred every few years (2009, 2012, and 2015) that was more than 200 mm (204.81 mm, 206.18 mm, and 216.92 mm, respectively). The displacement in other years increased by less than 100 mm. Another phenomenon was that the displacement during the step-wise deformation period (from May to September) contributed to the majority of the displacement in the whole year, especially from May to July, which contributed to more than 70% of the annual displacement. The maximum monthly displacement occurred in June or July each year, except 2015 (occurred in August). For example, the yearly displacement in 2012 was 206.18 mm; the displacement increment between May and July was 187.55 mm and occupied 91% of the whole year displacement. The maximum monthly rainfall occurred in June and was up to 164 mm. The reservoir level dropped between May and July 2012, and the cumulative rainfall rose to 349.73 mm. Thus, the time from May to July can be the critical early warning period for step-wise landslides. The deformation during this period was mainly controlled by reservoir water level decline and heavy rainfall.

### 3.2. Accumulated Displacement Decomposition

The monitored data of station ZG324 from January 2007 to July 2017 and from August 2017 to July 2018 were selected as training and testing data sets, respectively. An appropriate decomposition method is crucial in establishing a landslide displacement prediction model. Several methods have been used in accumulated displacement decomposition, as mentioned in the introduction, and each has advantages and disadvantages. Zhu et al. [54] and Fu et al. [55] have demonstrated that CEEMD is an effective method for reconstructing landslide displacement, with the advantages of a high stability and complete decomposition. Therefore, the CEEMD method was adopted here to decompose accumulated displacement into trend term and periodic term displacements.

**Figure 11:**
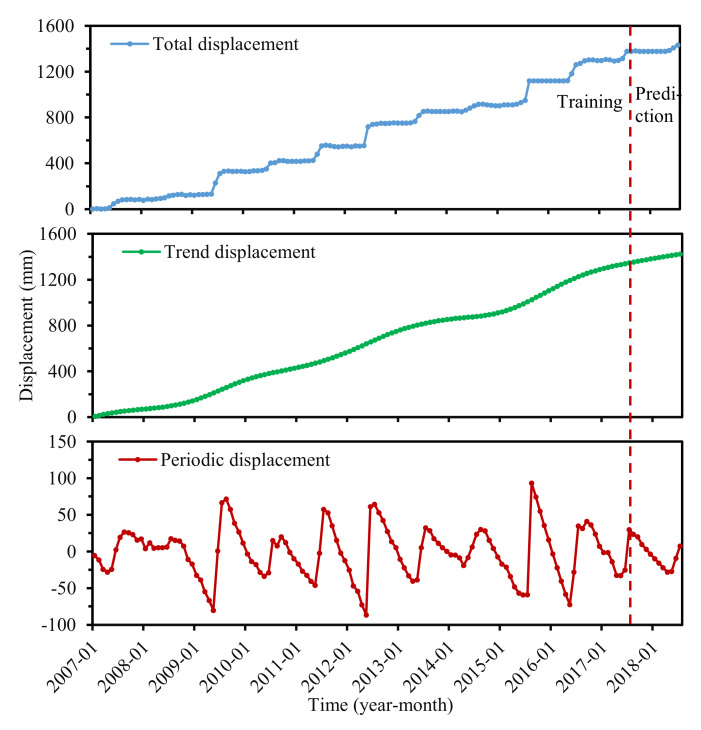
Displacement decomposition at ZG324.

In the training of the forecast model, we tested 200 trials and set the standard deviation of the added white noise in each ensemble to 0.25. We used the CEEMD to decompose the accumulated displacement into several IMFs and a residual, while the residual represented a trend component. Subsequently, we can obtain the periodic displacement by summing up all of the IMFs or subtracting the trend term from the accumulated displacement. Figure 11 displayed the trend and periodic components of ZG324 in the Baijiabao landslide.

### 3.3. Trend Displacement Prediction

**Figure 12:**
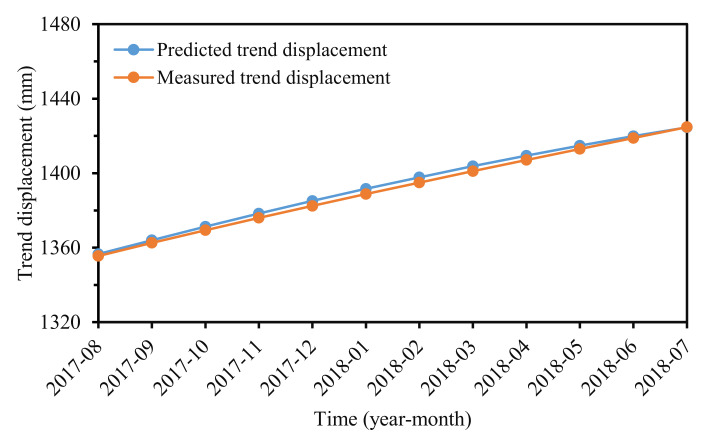
Predicted and measured trend displacement.

Controlled by “internal” conditions, the trend displacement increases monotonically with time [23]. Some researchers forecasted trend displacement by fitting the displacement–time curve, and a polynomial was commonly used [33,37]. However, a single function can be insufficient to fit the curve properly [10]. A univariate GRU model was adopted to forecast the trend displacement in this study, and the established model achieved an excellent prediction performance (Figure 12). The prediction results of RMSE, MAPE, and R^2^-values were 2.09 mm, 0.14%, and 0.9984, respectively.

### 3.4. Periodic Displacement Prediction

#### 3.4.1. Triggering Factors Selection

Triggering factors selection is essential to guarantee the accuracy of a displacement predictor. According to the monitoring data of the Baijiabao landslide (Figure 9 and Figure 10), rainfall and reservoir water level fluctuation are two major factors triggering its step-wise deformation. Selby [56] proposed that the evolutionary state of landslides was also an influential factor in the dependence of the movement on external factors. By referring to the research [9,25,31,36] and our previous work [42], seven candidate triggering factors were considered here.

Gray relational analysis (GRA) was used to check the degree of correlation between the periodic displacement and candidate triggering factors [57]. In GRA, we chose periodic displacement and candidate triggering factors as primary sequence and sub-sequences, respectively. All the sequences were normalized in the following way:

(22) $$X_{k}{\left(i\right)}^{\prime }=X_{k}\left(i\right)/\frac{1}{n}\overset{n}{\underset{i=0}{\sum }}X_{k}\left(i\right)$$

where i=0,1,⋯,n; k=0,1,⋯,m; *n* is the number of data points; m is the number of candidate triggering factors. The correlation coefficients were thus obtained by Equation (23):

(23) $$\delta \left((x_{0}{\left(i\right)}^{\prime },x_{k}{\left(i\right)}^{\prime }\right)=\frac{p+\rho q}{\left|X_{k}{\left(i\right)}^{\prime }-X_{0}{\left(i\right)}^{\prime }\right|+\rho q}$$

(24) $$p=\min_{k}\min_{i}(X_{k}{\left(i\right)}^{\prime }-X_{0}{\left(i\right)}^{\prime })$$

(25) $$q=\max_{k}\max_{i}(X_{k}{\left(i\right)}^{\prime }-X_{0}{\left(i\right)}^{\prime })$$

where ρ is the resolution coefficient and is usually set to 0.5.

The grey relational grade (GRG) was adopted to evaluate the correlation between variables, and was calculated by Equation (26):

(26) $$r\left(x_{0},x_{i}\right)=\frac{1}{n}\overset{n}{\underset{k=1}{\sum }}\delta \left((x_{0}{\left(i\right)}^{\prime },x_{k}{\left(i\right)}^{\prime }\right)$$

The GRG values vary from 0 to 1, with GRG values above 0.6 indicating a strong correlation between variables. The results were summarized in Table 1. GRG values between all the variables were above 0.6, suggesting that the candidate triggering factors can be used as the input of the prediction model.

#### 3.4.2. Establishment of the Prediction Model

The training dataset was divided into training and validation sections, and they accounted for 70% and 30% of the total [9,35]. The triggering factors and periodic displacement were normalized to [−1, 1], and they were used as the input sequence and output sequence of the models, respectively. In this experiment, all the models used in the paper were implemented on MATLAB R2021a software, where the ML toolbox and deep ML toolbox were used. The GRU model had three layers: two were GRU layers, and the other one was a hidden layer. In the established GRU model, the number of hidden units was 200. The values of maximum epochs, minimum batch size, and initial learning rate were 250, 10, and 0.05, respectively. Those parameters of LSTM were 250, 1, and 0.01, respectively. In the RF model, the number of predictors and trees were 5 and 10, respectively.

**Figure 13:**
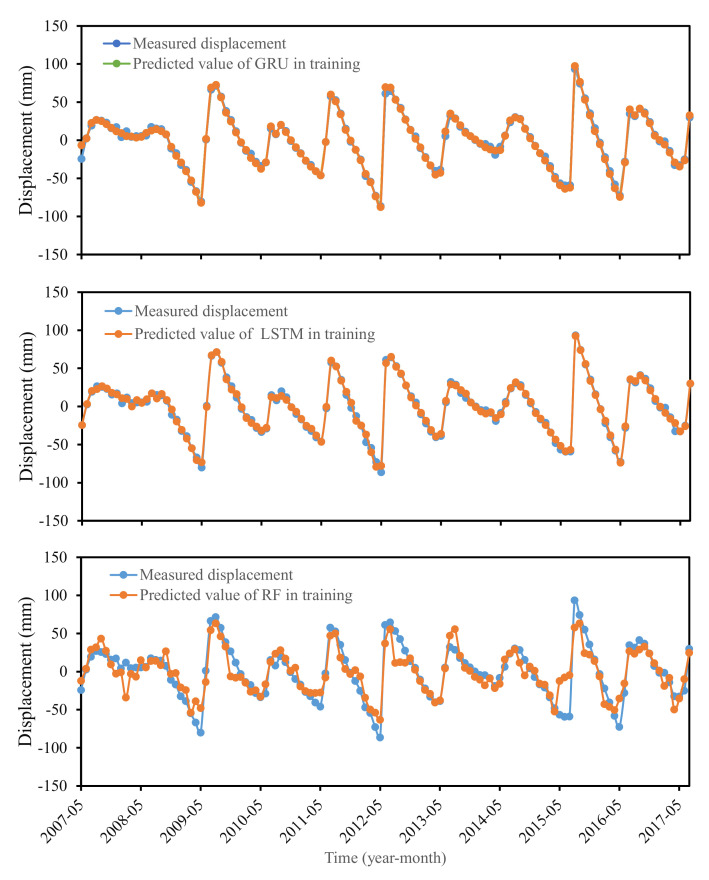
Measured and predicted displacements of GRU, LSTM, and RF models in training.

The predicted values of GRU, LSTM, and RF models in the training process were shown in Figure 13. The prediction accuracy of the trained models was shown in Table 2. It indicated that the predicted displacements fitted well with the measured displacement in the trained LSTM and GRU models and were more satisfied than the RF model.

#### 3.4.3. Predicted Periodic Displacement

**Figure 14:**
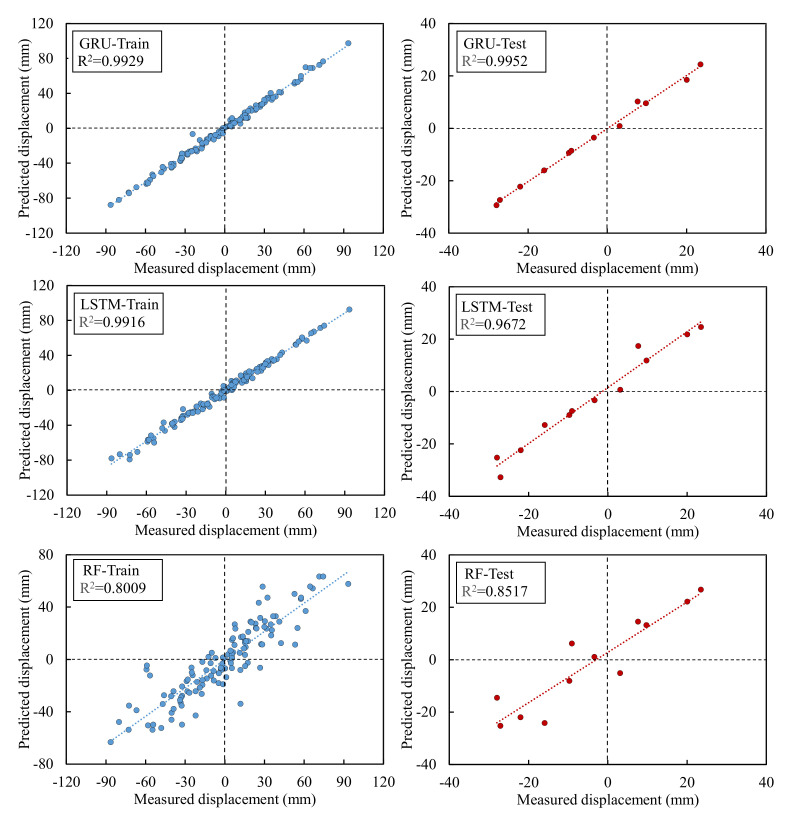
Training and prediction process of each model.

**Figure 15:**
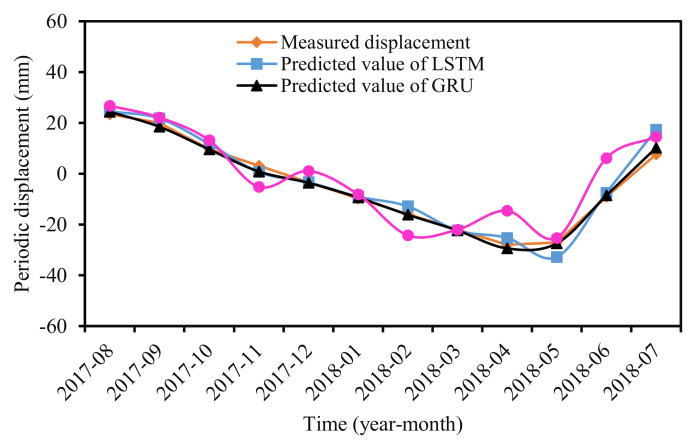
Predicted and measured periodic displacement.

Figure 14 and Figure 15 compared the measured and predicted periodic displacement at locations ZG324 using the GRU, LSTM, and RF models. The prediction accuracy of each model was summarized in Table 3. The GRU model gave the best agreement with the measured values in the three models, with RMSE, MAPE, and R^2^ values of 1.21 mm, 11.87%, and 0.9952. Another deep ML method—LSTM—showed a lower prediction accuracy than the GRU model. Its RMSE, MAPE, and R^2^ were 3.67 mm, 26.67%, and 0.9672, respectively. Compared with the two deep ML methods—LSTM and GRU—the ensemble model RF did not demonstrate a satisfied prediction performance, and the accuracy factors were 7.35 mm, 69.84%, and 0.8517.

The predicted displacements of GRU and LSTM aligned well with the measured displacement, including in the critical early warning period of the step-wise landslides (from May to July). During May to July 2018, the reservoir water level decreased from 160.39 m to 145.33 m, and the cumulative precipitation rose to 397.83 mm. The above two influencing factors caused the displacement to increase sharply. Several local peaks existed in the curve of the predicted results for the RF model. The error of each prediction time point (each month) was distributed disorderly.

It should be noted that the GRU model showed a better prediction performance than the LSTM and RF models on the whole rather than at every time point. For example, for the displacement prediction of March, 2018, the absolute error (AE) and relative error (RE) of the GRU model were 0.38 mm and 1.72%, whereas the indicators of the RF model were 0.27 mm and 1.25%.

### 3.5. Stochastic Displacement Prediction

**Figure 16:**
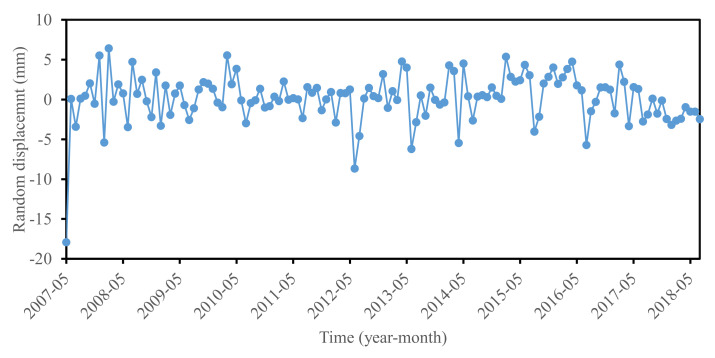
Stochastic displacement at ZG324.

According to displacement component composition, stochastic displacement can be obtained by removing the trend term and the periodic term from the accumulated displacement series. The results were shown in Figure 16, which indicated that stochastic displacement varied with time disorderly.

**Figure 17:**
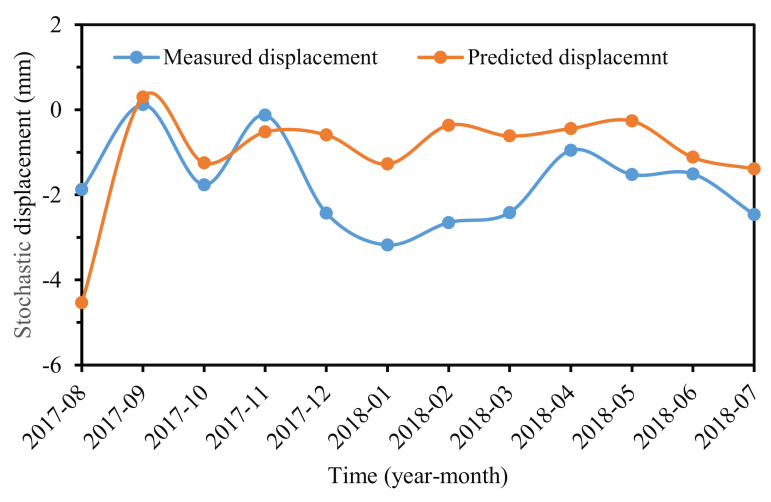
Predicted and measured stochastic displacement.

In this paper, the stochastic displacement of the Baijiabao landslide was trained and predicted by a multivariate GRU model. All of the impact factors and stochastic displacements were converted to a [−1, 1] format in sample data preprocessing. The prediction results were shown in Figure 17. The RMSE, MAPE, and R^2^ values were 1.48 mm, 94.36%, and 0.0793, respectively. The prediction accuracy was not satisfied, whereas the whole variant trend between the predicted value and measured stochastic displacement was identical.

### 3.6. Accumulated Displacement Prediction

**Figure 18:**
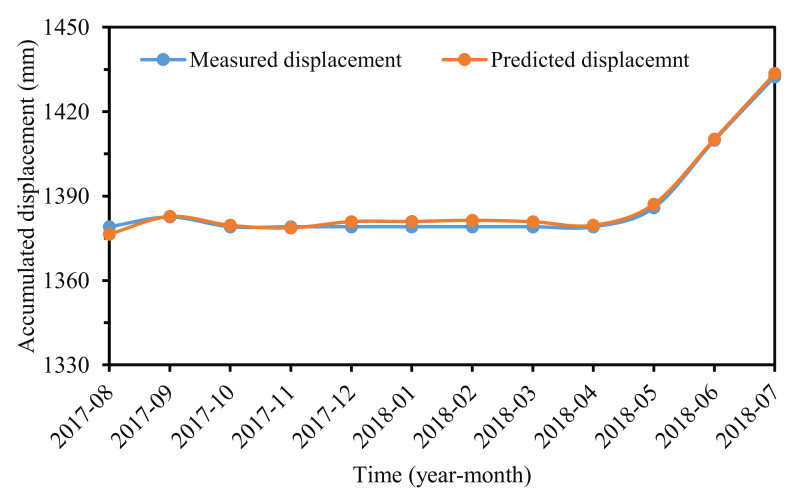
Predicted and measured accumulated displacement.

According to the accumulated displacement composition, the total displacement can be obtained by making the sum of the predicted trend and periodic and stochastic displacements. Figure 18 showed that the predicted accumulated displacements compared well with the measured displacement. The RMSE, MAPE, and R^2^ values were 1.48 mm, 0.09%, and 0.9936.

## 4. Discussion

It is critical to forecast periodic displacement accurately in the prediction of accumulated displacement for landslides with step-wise deformation [23]. Multiple ML methods have been proposed and adopted in the periodic displacement prediction, such as BPNN, ElM, SVM, RF, etc. The evolution process of landslides is a dynamic, complex, and nonlinear system. With the advantages of handling complex nonlinear problems and considering the dynamic evolution, a deep dynamic model—GRU—is thus selected to predict landslide periodic displacement.

The performance of the model was validated with the observations of the Baijiabao landslide. Another two popular models, LSTM and RF, were adopted for comparison. The results showed that GRU achieved the best prediction accuracy in the three models. Compared with RF, GRU has the ability to establish connections between adjacent time steps, and this structure contributes to improving the prediction performance of the models. Compared with LSTM, GRU has a simpler structure and fewer hyperparameters. Thus, it can be easier to establish a well-trained GRU model and achieve a better prediction accuracy. It should be noted that though GRU indicated a higher prediction accuracy for one monitoring point in the Baijiabao landslide, this does not mean that the model applies to all landslides. The limitation of generalization inherent in the GRU model makes it difficult to predict all cases accurately. Such a limitation exists in all models [37]. To deal with this problem, ensemble models can be established by combining several models with different weights of the individual model [58]. In addition, switched prediction methods can be adopted to select the appropriate individual prediction model from several candidate models for a landslide [59].

Although the GRU model achieved an encouraging prediction accuracy, it has some drawbacks. One drawback is that the GRU uses the stochastic gradient descent optimization algorithm to update weights, which risks falling into local optimization [60]. Another drawback is that the deep GRU model demands a larger dataset size than conventional ML models [10]. The monitoring frequency is one time per month for the GPS data used in the Baijiabao case. It may take years to obtain enough data for the prediction model. If not enough training samples are available, the neural network cannot be fully trained, and therefore the prediction accuracy of the model will be affected. This drawback of GRU places a higher requirement on the monitored data of landslide deformation.

The stochastic displacement is induced by some stochastic factors, including earthquakes, wind load, and vehicle load, which make it a disordered series (Figure 14). This feature contributes to the difficulty in stochastic displacement accurate prediction. Little research on stochastic displacement prediction has been reported [33]. If a slope is marginally stable or even unstable, a slight stochastic “load” can lead to disequilibrium and intense deformation. The ignorance or underestimation of stochastic displacement may make landslide planners carry out nothing, thus increasing the possibility of landslide accidents. In this paper, stochastic component displacement was considered in accumulated displacement prediction. The stochastic displacement was determined by deducting the trend and periodic displacements from accumulated displacement, and was predicted by a multivariable GRU model. The prediction performance was unsatisfactory due to the varied, ever-present, and unquantifiable stochastic factors. The work is still a helpful experiment for understanding landslide displacement components and serves as an early warning for landslides. One should consider methods to develop optimal models for predicting stochastic displacement in the future [37].

The temporal prediction of landslides is one of the main components of early warning systems [61]. Empirical methods based on the trend of landslide rate and semi-empirical practices based on the displacement rate and acceleration can provide an estimation of landslide failure time [62]. In addition, multiple parameters relating to displacement, such as the displacement rate, displacement acceleration, and tangential angle, have been proposed as thresholds to suggest a probable failure, although these approaches cannot provide a time frame for such an occurrence [63]. Realizing the temporal prediction of landslides at slope-scale based on relating the displacement would require a deeper dissertation in future work.

## 5. Conclusions

Displacement prediction is a vital and economic measure for landslide risk reduction and always emphasizes landslide research. This paper decomposed accumulated displacement into different displacement components by CEEMD. A univariate GRU model and a multivariable GRU model were used to predict the trend and stochastic displacements. A multivariable GRU model was used to establish a predictor for periodic displacement prediction, and two other popular ML models—LSTM and RF—were adopted for comparison. The predicted accumulated displacement was gained by the superposition of the three predicted displacement components. The results showed that predictors of deep ML methods—GRU and LSTM—had a higher prediction accuracy than the RF model in the studied case, which revealed the superiority of deep ML methods in long time series prediction. Both as deep ML methods, the GRU model achieved a better prediction performance than the LSTM model. One main reason is that the GRU algorithm has fewer hyperparameters to be trained in the model establishment than the LSTM algorithm. A prediction model with the structure of CEEMD—univariate GRU (trend displacement), multivariable GRU (periodic displacement), and multivariable GRU (stochastic displacement)—was proposed and achieved an encouraging prediction performance. The proposed model can be a potential tool for landslide risk reduction in the dam reservoir.

## Figures and Tables

**Table 1:** Candidate factors for the periodic displacement of Baijiabao landslide.

Inputs 1–7	Grey Relational Grade (GRG)
Input 1: the 1-month antecedent rainfall	0.68
Input 2: the 2-month antecedent rainfall	0.68
Input 3: average reservoir elevation in the current month	0.69
Input 4: change in reservoir level over the last month	0.72
Input 5: the displacement over the past month	0.71
Input 6: the displacement over the past two months	0.70
Input 7: the displacement over the past three months	0.69

**Table 2:** Prediction accuracy of the trained models.

Model	RMSE (mm)	MAPE (%)	R^2^
GRU	3.12	21.22	0.9929
LSTM	3.67	30.04	0.9916
RF	15.95	109.21	0.8009

**Table 3:** Prediction accuracy of periodic displacement.

Model	RMSE (mm)	MAPE (%)	R^2^
GRU	1.21	11.87	0.9952
LSTM	3.67	26.67	0.9672
RF	7.35	69.84	0.8517

## Data Availability

Some or all data, models, or code that support the findings of this study are available from the corresponding author upon reasonable request.
